# Usability and Effectiveness of an Individualized, Tablet-Based, Multidomain Exercise Program for People With Dementia Delivered by Nursing Assistants: Protocol for an Evaluation of the InCoPE-App

**DOI:** 10.2196/36247

**Published:** 2022-09-26

**Authors:** Bettina Barisch-Fritz, Jelena Bezold, Andrea Scharpf, Sandra Trautwein, Janina Krell-Roesch, Alexander Woll

**Affiliations:** 1 Institue of Sports and Sports Science Karlsruhe Institute of Technology Karlsruhe Germany

**Keywords:** institutionalization, institutionalized, sport, physical activity, fitness, exercise, dementia, digital application, cognitive performance, physical performance, cognitive function, physical function, cognitive decline, nursing home, long-term care, usability, effectiveness, mHealth, mobile health, health app

## Abstract

**Background:**

The COVID-19 pandemic has had drastic consequences on everyday life in nursing homes. Limited personnel resources and modified hygiene and safety measures (eg, no external exercise instructors, no group settings) have often led to interrupted physical exercise treatments. As a consequence, people with dementia benefiting from individualized exercise programs are affected by the pandemic’s impact.

**Objective:**

Our goal is to develop an easily applicable mobile application (Individualized Cognitive and Physical Exercise [InCoPE] app) allowing nursing assistants to test cognitive function and physical performance and subsequently train people with dementia through a multidomain, individualized exercise program.

**Methods:**

We will evaluate the usability and effectiveness of the InCoPE-App by applying a mixed method design. Nursing assistants will use the InCoPE-App for 18 weeks to assess the cognitive function and physical performance of 44 people with dementia every 3 weeks and apply the individualized exercise program. We will record overall usability using questionnaires (eg, Post-Study System Usability and ISONORM 9241/10), log events, and interviews. Perceived hedonic and pragmatic quality will be assessed using the AttrakDiff questionnaire. Effectiveness will be evaluated by considering changes in quality of life as well as cognitive function and physical performance between before and after the program.

**Results:**

Enrollment into the study will be completed in the first half of 2022. We expect an improvement in the quality of life of people with dementia accompanied by improvements in cognitive function and physical performance. The usability of the InCoPE-App is expected to be rated well by nursing assistants.

**Conclusions:**

To date, there is no scientifically evaluated app available that enables nursing assistants without expertise in sports science to deliver an individualized exercise program among people with dementia. A highly usable and effective InCoPE-App allows nursing assistants to test cognitive function and physical performance of people with dementia and, based thereon, select and deliver an appropriate individualized exercise program based on the cognitive and physical status of an individual, even in times of a pandemic.

**Trial Registration:**

German Register of Clinical Trials DRKS00024069; https://www.drks.de/drks_web/navigate.do?navigationId=trial.HTML&TRIAL_ID=DRKS00024069

**International Registered Report Identifier (IRRID):**

DERR1-10.2196/36247

## Introduction

The COVID-19 pandemic has had drastic consequences for all areas of everyday life. In many nursing homes, routine processes had to be changed. Some activities and treatments were reduced or, in areas, canceled. Hygiene and safety measures (eg, no external exercise instructors, no group settings) and the increased workload for nursing assistants led to suspending physical exercise programs and activities, especially when delivered in a group setting. The potential consequences for nursing home residents include reduced general activity accompanied by decrease in cognitive function and physical performance and a considerable loss of an individual’s quality of life. Furthermore, these COVID-19–induced changes may also have an economic impact and may increase care expenses in the long term.

Dementia is present in up to 50% of nursing home residents [1]. The clinical symptoms of this neurodegenerative disease include, but are not restricted to, decreased cognitive skills and reduced physical performance (eg, balance and mobility) [2,3]. Many people with dementia experience a rapid progression of the disease [1]. Thus, people with dementia might be seriously affected by COVID-19–induced reduction of physical or mentally stimulating activities and similar treatments (eg, physiotherapy, ergo therapy, social interactions) in nursing homes.

In light of the absence of medication to cure dementia, physical activity is one of the major nonpharmacological interventions that has become very important in treating people with dementia. Previous reviews have mainly reported positive effects of physical activity on cognitive but also physical outcomes in people with dementia [4-8]. However, conclusive evidence is still lacking, mainly due to limited numbers of high-quality studies and large heterogeneity in methods and exercise program parameters (eg, scope, duration, intensity, contents, settings). Of note, an individual’s prior exercise experiences and preferences may also contribute to the success of an intervention. These factors, combined with the older age of people with dementia as well as the different stages and degrees of impairment, result in the highly heterogeneous samples often found in research studies. To elicit the potential effects of physical activity interventions in people with dementia, we have previously argued that individualization of the exercise program may thus be crucial [9]. To the best of our knowledge, individualized exercise programs are not sufficiently included in routines of nursing homes, and optimal or successful ways of distribution have not been reported in prior research studies.

Fundamental changes in health practice are driven by recent developments in interactive health technologies that promote health and manage illness [10]. These technologies have been reported to elicit beneficial health outcomes in various settings (eg, physical activity coaching app for breast cancer [11], Partners in School Asthma Management for inner-city elementary school children [12]). The number of digital health apps has increased exponentially, whereas the number of publications addressing their usability evaluation has remained at a low level [13]. There is still a lack of reports on the development and usability processes during app development in digital health settings. More than 95% of apps available today have not been scientifically tested, and the limited number of controlled trials of mobile technology interventions reported only modest effects [14]. However, usability is a crucial component of good practice in the development of digital apps [15]. End users’ needs are particularly important in digital health applications to ensure that individuals affected by any health issue can use the intervention appropriately, which may in turn lead to greater acceptance and thus efficiency of the intervention [16]. The ISO standard 9241-11 defines usability as the extent to which a product can be used to achieve the specified goals with effectiveness, efficiency, and satisfaction [17]. Another standard on software quality, ISO 25010, includes attributes for usability such as understandability, learnability, operability, and attractiveness [18].

Our team will develop a tablet-based mobile app aimed at supporting nursing assistants to (1) test the cognitive function and physical performance of people with dementia and (2) subsequently train people with dementia. The training is a multidomain, individualized exercise program that will be adjusted to the individual’s current physical and cognitive performance with the focus of improving physical and cognitive performance in the short and long term. In times of COVID-19, this is a novel and innovative approach that allows nursing homes to maintain a physical activity routine while complying with hygiene and safety measures, as no external exercise instructor is needed. The individualization approach used in this study in combination with the direct applicability in the setting of nursing homes is promising in terms of the individual benefit of the exercise program on physical and cognitive performance as well as quality of life. The aim of the present study is to evaluate the usability and effectiveness of the individualized exercise program provided by the Individualized Cognitive and Physical Exercise (InCoPE)-App.

## Methods

### Study Design

**Figure 1:**
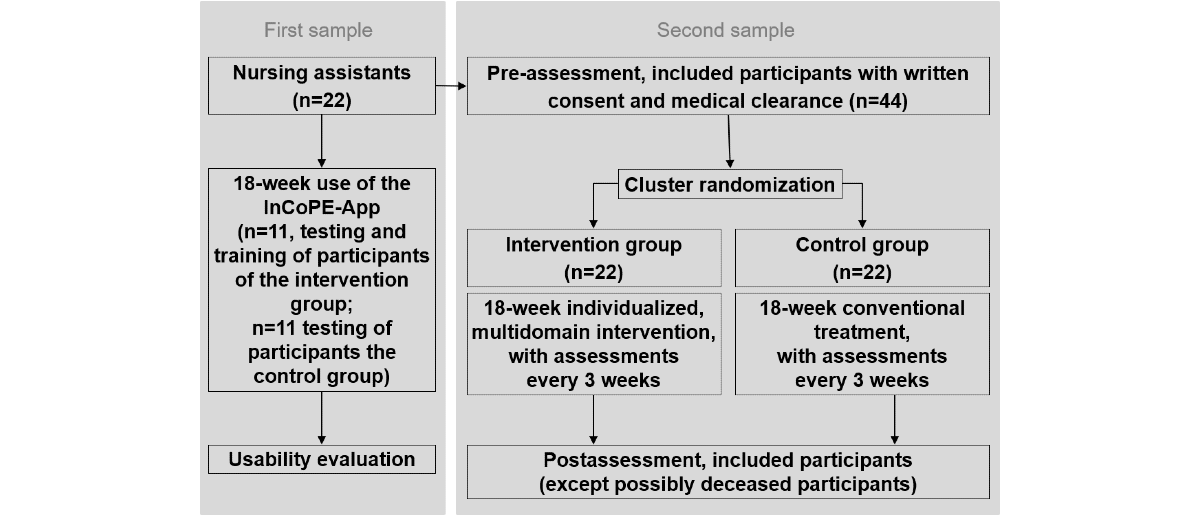
Flow of participants. InCoPE: Individualized Cognitive and Physical Exercise.

This study combines the evaluation of the usability of the InCoPE-App with the effectiveness of the individualized exercise program concerning cognitive function, physical performance, and quality of life. Thus, a mixed methods design with different methodological components will be used. The evaluation of the usability and effectiveness of the InCoPE-App will be based on the use of the app for a duration of 18 weeks in a nursing home setting. This approach represents a field study and will be carried out as a cluster-randomized controlled trial in which the InCope-App will be used with people with dementia and the individualized, multidomain exercise program will be delivered by nursing assistants with the help of the app. The nursing homes will be cluster randomized, and each nursing assistant will subsequently recruit at least 2 people with dementia (see Figure 1).

### Ethical Approval

The study is conducted in accordance with the Declaration of Helsinki. The study was approved by the Ethics Committee of the Karlsruhe Institute of Technology (Karlsruhe, Germany).

### Participants

To evaluate the InCoPE-App in terms of usability and effectiveness, 2 different study samples are required (see Figure 1). The first sample will include the future users of the InCoPE-App (ie, nursing assistants). To this end, initial contact will be established through the heads of care facilities in southwestern Germany who suggest potentially interested nursing assistants, who will subsequently receive detailed information about the aims and the content of the study. The following inclusion criteria must be fulfilled: (1) willingness to participate in the training to familiarize themselves with the InCoPE app, (2) command of the German language as the InCoPE-App is only available in German at this time, and (3) employment at the nursing home for at least 18 weeks. External employees (eg, physical therapists) will be excluded.

The second sample will include participants with primary dementia living in nursing homes. This sample will be drawn to evaluate usability and trends toward the effectiveness of the individualized exercise program as delivered by the InCoPE app. Participants in the second sample will be selected by nursing assistants. Before entering the study, written consent by participants or their legal guardians will be obtained. Prior to the intervention, a clearance certificate from the participant’s general practitioner will be collected, which will also include information about the dementia diagnosis and other pertinent information such as medication intake and comorbidities. Additional inclusion criteria are (1) Alzheimer disease, vascular dementia, or other primary dementia; (2) mild to moderate stage of dementia (Mini-Mental State Examination [MMSE]: 10-24); (3) age older than 65 years; and (4) walking ability of at least 10 meters with or without a walking aid. Exclusion criteria include (1) secondary dementia, (2) other severe cognitive impairments, (3) other severe neurological conditions, (4) other severe acute diseases, and (5) severe motor impairments.

### Intervention

The individualized exercise program for people with dementia is based on earlier work by this research group and includes a combination of cognitive and physical exercises [19]. The included exercises were further differentiated to ensure a higher degree of individualization following the idea of individualized medicine [20]. The individualized exercise program is embedded in the InCoPE-App, which will be utilized by nursing assistants. The InCoPE-App will be installed on a tablet device (Android), and nursing assistants will be trained in the appropriate use of the app in order to test and instruct people with dementia. The training comprises theoretical online modules for self-study (eg, theoretical basics of physical exercise with people with dementia, objective testing of physical and cognitive performance in people with dementia, first steps with the InCoPE-App) and 2 face-to-face meetings (between project members or research staff and nursing assistants) for the usage of the InCoPE-App. The InCoPE-App contains detailed photographic materials and descriptions to offer visual support in the execution of the proposed tests and exercises.

The tests assessing motor and cognitive performance included in the InCoPE-App are based on published recommendations and were used with people with dementia before [21,22]. On the basis of cluster analysis in a large sample of people with dementia who participated in our group’s previous randomized controlled trial [19], we identified 4 homogeneous subgroups of people with dementia [23] who were also considered when we designed the InCoPE-App: participants with (1) below-average motor and cognitive performance, (2) average cognitive performance and above-average motor performance, (3) above-average cognitive and motor performance, and (4) above-average cognitive performance and below-average motor performance. The initial testing of physical and cognitive performance included in the InCoPE-App will be used to determine whether an individual belongs to cluster 1, 2, 3, or 4. Testing will be repeated every 3 weeks during the 18-week intervention period to allow the InCoPE-App to regularly adjust the duration, intensity, and contents of the cognitive and physical exercises based on the individual’s needs. We anticipate that the desired physical and cognitive adaptation to the exercise program will be achieved by gradually increasing exercise difficulty and intensity (eg, number of repetitions).

For each individual, the content of the individualized exercise program will vary based on the cluster categorization. For example, a person in cluster 1 will receive recommendations to complete exercises that focus on increasing balance, mobility, and strength as well as cognitive stimulation. In contrast, a person in cluster 4 will likely undergo exercise with a more pronounced focus on balance, mobility, or strength regardless of the degree of cognitive stimulation. Furthermore, promotion of endurance is part of every training session. The differentiation of each exercise is regulated via several degrees of difficulty and intensity. Moreover, additional cognitive input is subdivided into 3 levels of difficulty. Training sessions will take place twice per week on nonconsecutive days for 60 minutes. These 60 minutes are divided into a ritualized warm-up, first combination of exercises, short break with ritualized exercises for balance, second combination of exercises, and cooldown. Pure training duration will be about 40 minutes. The ritualization of training sequences will give the people with dementia a sense of security.

### Outcomes

#### Primary Outcomes

The primary outcomes were defined along the overall aim to evaluate the usability and effectiveness of the individualized exercise program delivered via the InCoPE-App. In Table 1, the questionnaires and measures for usability, quality of life, and overall cognition as well as methods to evaluate executive function and physical performance are listed.

**Table 1:** Primary outcomes.

Dimensions	Assessments	Pre	Post	Implemented in the InCoPE^a^-App
Overall usability	Post-Study System Usability Questionnaire (PSSUQ) version 3 [24]	No	Yes	No
Overall usability	ISONORM, a questionnaire that operationalizes the 7 criteria of EN ISO 9241-10 [25]	No	Yes	No
Perceived hedonic and pragmatic quality	AttrakDiff 2 [26]	No	Yes	No
Quality of life	German Quality of Life-Alzheimer’s Disease (QOL-AD) [27]	Yes	Yes	No
Global cognition	Mini-Mental State Examination Test (MMSE) [28]	Yes	Yes	Yes
Executive function	Digit span [29]	Yes	Yes	Yes
Mobility	Timed Up & Go (TUG) test [30]	Yes	Yes	Yes
Mobility	6-meter walking test (6MWT) [31]	Yes	Yes	Yes
Function and strength of lower limbs	Modified 30-second chair stand test (CST) [32]	Yes	Yes	Yes
Balance	Frailty and Injuries: Cooperative Studies of Intervention Techniques-subtest 4 (FICSIT-4) [33]	Yes	Yes	Yes

^a^InCoPE: Individualized Cognitive and Physical Exercise.

Each nursing assistant will use the app to deliver the individualized exercise program within the 18-week intervention to 2 people with dementia. The overall usability will be assessed after the intervention using the Post-Study System Usability Questionnaire (PSSUQ) [24]. The PSSUQ gives insight into user satisfaction with a system (eg, system usefulness, information quality, and interface quality). This questionnaire can be considered a useful tool for field studies [24]. We will use PSSUQ version 3, which is comprised of 19 items such as system usefulness, information quality, and interface quality rated on a 7-point Likert scale (1: strongly agree to 7: strongly disagree) [24,34]. Furthermore, usability will be assessed using the ISONORM questionnaire [25], which covers the implementation of the 7 defined criteria based on the International Ergonomics Standard DIN EN ISO 9241-110. This questionnaire operationalizes the 7 criteria of the EN ISO 9241-10 and was developed to evaluate software development [35]. The completion of the questionnaire takes 10 minutes, and bipolar statements from 35 items are assessed using a 7-point Likert scale (1: very negative to 7: very positive) [35]. The perceived hedonic and pragmatic quality of the InCoPE-App will be assessed using the AttrakDiff 2 questionnaire [26]. This questionnaire evaluates the participants’ perceptions of the InCoPE-App by means of semantic differentials.

Examination of the effects of the InCoPE-App on physical and cognitive performance in people with dementia will be determined based on the listed variables in Table 1. The primary outcomes will be assessed before and after the 18-week intervention. To assess quality of life, the 13-item caregiver-administered version of Quality of Life-Alzheimer’s Disease (QOL-AD) [27] will be applied. The QOL-AD is a valid and reliable tool (internal reliability, α=0.88-0.89; test-retest reliability for caregivers, 0.92) [27]. Global cognition will be assessed with the MMSE [28]. The questionnaire will be implemented in the InCoPE-App. The MMSE easily assesses 7 areas of cognitive function, with sufficient test-retest reliability (0.80-0.95) [28,36]. To assess executive function, the Digit Span Test [29] will be administered. The Digit Span Test consists of 2 parts: forward and backward. Participants will be asked to repeat a sequence of 3 to 9 digits forward and of 2 to 8 digits backward. The InCoPE-App will be used to document the given answers. The Digit Span is a reliable and valid test [37].

The Timed Up & Go (TUG) test will be conducted by asking participants to rise from a chair, walk 3 meters, turn around, and then go back and sit down again on the chair [30]. Nursing assistants will instruct participants while using the InCoPE-App to assess the time taken by each participant. Time is measured from the initial impulse to stand up until participants are seated again. Everyday walking aids are allowed. For the 6-meter walk test (6MWT) [31], participants will be asked to walk from one side of the room to the other side, where the distance of 6 meters will be marked using start and finish lines on the floor. When participants cross these lines, the time required to walk 6 meters will be measured using the InCoPE-App. In front of the starting line and behind the finish line, participants will have about 2 meters for acceleration and deceleration. If needed, participants are allowed to use their walking aids. Strength and function of lower limbs will be assessed using the modified 30-second chair stand test (30s CST). For the modified 30s CST, participants will be asked to stand up from a chair (height 46 centimeters, with armrests) as often as possible for 30 seconds. The modified version allows the use of armrests [32], which is essential for the majority of older adults with dementia to safely perform this test. While testing lower extremities, no walking aids are allowed. Static balance will be determined using the Frailty and Injuries: Cooperative Studies of Intervention Techniques- subtest 4 (FICSIT-4) [33], in which participants are asked to perform 4 different standing positions (Romberg, semitandem, tandem, and single leg) for 10 seconds without walking aids or other assistance. The FICSIT-4 performance is rated on a scale of 0 to 5 points. If participants cannot hold the first position for at least 3 seconds, a score of 0 is given. In contrast, participants receive a score of 5 if they are able to stand in the most difficult (ie, single leg) position for at least 10 seconds.

The chosen tests for physical performance are considered reliable tests in a geriatric setting. Among people with dementia, the intraclass correlation coefficients for test-retest reliability are 0.79-0.82 for FICSIT-4, 0.83-0.89 for 6MWT, 0.78-0.88 for the modified 30s CST, and 0.72-0.99 for the TUG test [38,39]. The 95% minimal detectable changes are 58.9%-71.1% for FICSIT-4, 31.6%-41.5% for 6MWT, 33.2%-45.7% for the modified 30s CST, and 15.8%-39.6% for the TUG test [38,39]. No information about content and construct validity is available in this setting.

#### Secondary Outcomes

Data used to determine the usability as well as the subcategory of feasibility will be assessed through multiple methods (see Table 2). These methods help to get deeper insight and can improve the reliability and validity of the findings [40]. We will combine quantitative (ie questionnaires, task completions, and log events) and qualitative (ie, interviews and field notes) methods to produce a rich data set, which is especially recommended in health informatics research [41,42].

**Table 2:** Secondary outcomes of the usability measures and logged events as well as requested information measures.

Content	Recording method
Field notes (eg, participation in tests and training)	Qualitative and quantitative analysis
Interviews on satisfaction	Qualitative analysis
Number of completed training sessions	Quantitative output of the InCoPE^a^-App
Number of successfully completed testing periods	Quantitative output of the InCoPE-App
Number of rejected exercises	Quantitative output of the InCoPE-App
Kind of rejected exercises	Quantitative output of the InCoPE-App
Mean and range of the actual duration of the tests and the training sessions	Quantitative output of the InCoPE-App
Number and content of telephone calls	Qualitative analysis
Number and content of emails	Qualitative analysis
Satisfaction with the training session	Request after training session
Participation of the participant	Request after training session

^a^InCoPE: Individualized Cognitive and Physical Exercise.

Several forms of field notes, such as participation of participants after each test and exercise session, will be documented during the whole intervention. Additionally, nursing assistants will be asked to submit feedback, and we will regularly get in touch with nursing assistants by phone calls and emails to identify possible problems that arise when delivering the individualized exercise program through the InCoPE app.

The logged events and other pertinent information (see Table 2) are a substantial part of the usability with their subgenre feasibility. These feasibility measures place focus on app contents instead of human-technology interface. The feasibility measures are used to assess practicality and satisfaction as well as acceptance of the individualized exercise program but also the respective exercises and the testing. To this end, variables aimed at the exercises, testing, and participation will be collected by analyzing logged events during the entire 18-week intervention.

Interviews will focus on satisfaction with the InCoPE-App. The open-ended approach of Georgsson and Staggers [43] will serve as reference to ask users about aspects of the InCoPE-App, ranging from good to poor usability. We will ask 3 questions: (1) What parts of the system did you think were well designed? (2) Which parts of the system did you think were inadequately designed? (3) Do you have any other comments about the system functions and regarding its usability? All interviews will be conducted on a guided basis and will be held in German. With informed consent of the participants, the interviews will be recorded using a voice recorder (Philips DVT2050, Eindhoven, Netherlands).

Additional secondary outcomes will be assessed to obtain a holistic view of the participants who will be trained with the InCoPE-App. These physical and sociodemographic variables and respective assessment tools are presented in Table 3.

**Table 3:** Secondary outcomes on physical and sociodemographic information.

Dimensions	Assessments	Pre	Post	Implemented in the InCoPE^a^-App
Grip strength	Balloon manometer [44]	Yes	Yes	Yes
Frailty	Standardized phenotype of frailty in older adults [45]	Yes	No	Yes
Pain	Single item	Yes	Yes	Yes
Sociodemographic data and medical information	Singe item	Yes	No	No
Body height and weight	Single item	Yes	No	Yes
Fear of falling	FES-I^b^ [46]	Yes	No	No

^a^InCoPE: Individualized Cognitive and Physical Exercise.

^b^FES-I: Falls Efficacy Scale International Version.

Grip strength is measured with a balloon manometer [44]. Participants sit on a chair without armrests and solid feet-to-floor contact. Participants will be instructed and verbally encouraged to squeeze the balloon as hard as possible. Both hands will be tested. While being tested, participants should keep the upper arm in contact with the trunk and flex the elbow 90°. Additionally, the wrist is held in a neutral position (thumb up). To examine frailty, the Fried Frailty Criteria will be used [45]. Body weight, self-reported exhaustion, grip strength, walking speed, and level of physical activity in the past 3 months will be assessed before and after the intervention.

Nursing assistants will ask the participants for their body height and weight and enter them directly into the InCoPE-App. Fear of falling will be measured with the German version of the Falls Efficacy Scale International Version (FES-I). The questionnaire consists of 16 items rated on a 4-point scale that are combined into a total score. High values indicate a high fear of falling. Several studies have reported this instrument’s high reliability and validity [46].

### Sample Size

The sample size for the number of nursing assistants who will evaluate the InCoPE-App with regard to usability was defined using a probabilistic model of problem discovery for formative user research [34]. We defined a sample size comprising 22 participants to have a 90% chance of observing with a probability of 0.1. In the second study phase, outcomes also include usability in addition to the effectiveness of the InCoPE-App with respect to adaptations in physical and cognitive performance in people with dementia. For this reason, each participant will recruit 2 appropriate residents with dementia in accordance with our inclusion and exclusion criteria. We anticipate that our calculated sample size (N=44: intervention group [IG], n=22; control group [CG], n=22) will be appropriate to examine potential trends toward effectiveness of the InCoPE-App.

This sample size allows detection of moderate effects calculated using an ANOVA with repeated measures for within and between interactions. The calculation of the sample size with G*Power version 3.1.9.2 (Heinrich Heine University of Dusseldorf, Germany [47]; effect size ηp² = 0.07, α error probability .05, power 0.95) resulted in 44 participants.

### Randomization and Concealment

The residents with dementia recruited by nursing assistants will be allocated to either the CG or IG. This allocation will be performed by cluster randomization as it is not possible to blind participants, nursing assistants, or study personnel regarding group allocation. Participants in the CG will only receive conventional treatment such as individualized medication, standard care, or therapeutic applications for 18 weeks. Participants in the IG will receive the individualized exercise program in addition to conventional treatment. To ensure equal conventional treatment for the IG and CG during the intervention, it will be continuously documented.

### Data Management

#### Patient Documentation

The flow of participants will be organized and documented with an Excel documentation file. The participating nursing assistants will be assigned a 2-digit ID (pseudonymization of the data). All personally identifiable information will be documented in a separate Excel file and will be stored separately. The residents with dementia trained by nursing assistants will receive a 3-digit ID, where the first digit will encode the assignment to IG or CG. The collection and storage of the data will be performed separately. Only selected members of the project team will have access to uncoded data. The InCoPE-App is not connected to the internet to ensure security of personal data during the study. After the end of the 18-week intervention, collected data will be transferred manually via cable and manually stored on a computer that is not connected to the internet.

The collection and retrieval of the data from the questionnaires focusing on usability will be done manually (paper and pencil) and via SoSciSurvey. The entered data will be checked for completeness, validity, and plausibility by a member of the research team.

#### Data Monitoring

This study represents a non-drug intervention focusing on health benefits among people with dementia while undergoing an individualized exercise program delivered by trained nursing assistants who use the InCoPE-App. We do not expect any harmful effects nor adverse events related to the individualized exercise program. All exercises that are part of the program have been used in people with dementia before, and all questionnaires used to examine usability are derived from validated instruments that have been widely used in prior research. The study is thus considered to have minimal to no risks for participants, and establishing a data monitoring committee is not required. An interim analysis is not planned.

### Statistical Methods

All analyses will be performed using SPSS 27.0 (IBM Corp, Armonk, NY). Prior to analysis, plausibility (eg, considering range and distribution) will be checked to minimize errors. The quantitative analysis comprises the evaluation of the usability questionnaires, which will be analyzed with respect to the corresponding guidelines and will be compared with representative data.

The baseline values of physical and cognitive performance will be compared between IG and CG using chi-square tests for categorical data, Mann-Whitney *U* tests for nonparametric variables, and t tests for continuous and normally distributed parameters. Normal distribution will be checked with the Shapiro-Wilk test. Means and standard deviations will be calculated for normally distributed data, and medians and interpercentile ranges will be calculated for non-normally distributed data. A 2-factor ANOVA with repeated measurement will be used to identify treatment effects. In addition, 95% confidence intervals and partial Eta² will be calculated. The log events will be summarized and analyzed descriptively. Potential effects among subgroups based on varying physical or cognitive performance, gender, fear of falling, and logged events (completed training sessions, average duration of the training sessions) will be investigated using chi-square tests and *t* tests.

Qualitative content analysis of the field notes will be conducted [48]. The interviews will be fully transcribed and checked for accuracy. The entire coding process will be independently performed by 2 scientists. The transcripts will be reviewed to identify meaningful content-related aspects. These aspects will be systematically described using a category system. Research questions and interview guidelines will be consulted to deductively derive the main categories. Subcategories will be inductively developed based on the findings (eg, by subsumption). The category system will be tested by 2 scientists who will independently test-code several parts of the qualitative material and subsequently adapt the categories and their definitions. The 2 coding systems will eventually be merged by consensual coding where consensus will be reached.

## Results

Enrollment for the study started in October 2021. We plan to complete the postassessments of the last included nursing home in April 2022. Results are expected to be available in the second half of 2022.

## Discussion

### Summary

Physical activity in addition to conventional treatment can have a beneficial impact on cognitive and physical performance as well as quality of life in people with dementia. Providing scientifically grounded and individualized, multidomain exercise programs is challenging within everyday nursing care. The digital application InCoPE-App enables nursing assistants to regularly test cognitive and physical performance and subsequently train people with dementia based on their individual performance levels. We expect that the individualization of the exercise program as well as regular use ensured by high usability will result in increased physical and cognitive performance as well as quality of life in people with dementia after the 18-week program delivered by the InCoPE app. The increase in cognitive and physical performance may further ease the care demands and needs of people with dementia and thus possibly reduce the workload of nursing assistants.

### Strength and Limitations

Through the innovative and timely development method and thorough evaluation and integration of users’ experiences [49], we expect an application that is highly usable and feasible in everyday care situations. The acceptance of the InCoPE-App will promote its regular usage and thus allow testing and training residents with dementia individually. The regular conduct of physical and cognitive tests that is implemented as an essential part of the app will help to document the progression of disease symptoms and can thus be regarded as an additional objective tool for clinical documentation, which is required on a regular basis in a nursing home setting. The individualization of the exercise program, which has been described as critically important in previous research [9,20], is the key innovative and novel component of the InCoPE-App.

It is unlikely but possible that usability of the InCoPE-App will be rated as low by nursing assistants. In this case, fundamental changes within the application will be required to increase acceptance and practicability, which may have a considerable impact on the effectiveness of the exercise program. There are risks from a technical point of view that might impede the usability of the InCoPE-App, such as possible failure of the tablets to store the data on each tablet. To lower this risk, we will provide the required tablet with the installed InCoPE-App for each participating nursing home. In addition, nursing assistants can contact the research team to report technical issues and get help at any time.

Another limitation is the rather small number of participating people with dementia. This sample size is sufficient to detect moderate within and between interactions. However, it might be too small to statistically detect small effects. The use of the InCoPE-App within everyday life allows us to gain information about everyday feasibility and suitability; thus, results can be more easily transferred to daily routine. 

### Dissemination

A manuscript with the results on usability and effectiveness based on the primary outcomes will be published in a peer-reviewed journal. Additional manuscripts focusing on the secondary outcomes of the InCoPE-App will be prepared and submitted for publication in peer-reviewed journals. Upon completion of the study and after publication of the primary manuscript, all anonymized and summarized data can be requested from the researchers at the Institute of Sports and Sports Science of the Karlsruhe Institute of Technology, Germany.

The InCoPE-App will be evaluated in this study with respect to usability and effectiveness. After this evaluation, the participating nursing homes are allowed to further use the app to train their residents with dementia. Based on this, a dissemination plan will be developed to provide more nursing homes with the app, which is highly relevant in 2 ways. First, the continuing pandemic requires nursing assistants and residents with dementia to abide by strict hygiene measures, which is difficult when conducting a group-based exercise program possibly delivered by external employees such as trainers or instructors. Second, apart from pandemic circumstances, individualized group exercise programs in nursing homes are often delivered by nursing assistants who are not sufficiently trained for this purpose. Thus, the overall effect of exercise programs in nursing home settings is often limited. The InCoPE-App addresses this gap as it enables nursing assistants to deliver a scientifically based and individually assigned exercise program to people with dementia without requiring exercise science expertise or excessive prior training. Implementing the app in a nursing home setting may also be associated with economic advantages as additional employees with exercise science background to carry out a dementia-specific individualized exercise program may not need to be hired. One can conclude that the additional temporal expense might be balanced and the benefit of each participant can outweigh the expenses. The exact time and personnel resources required for implementation of the InCoPE-App will be assessed in the context of this study. This information can be used as key data to establish the InCoPE-App in other nursing homes.

### Conclusion

The InCoPE-App is a novel, innovative, tablet-based intervention that allows nursing assistants to individually test and train people with dementia with regard to physical and cognitive capacities while meeting the hygiene and safety measures in place during the current COVID-19 pandemic. The InCoPE-App can be used by nursing assistants without prior exercise experience as it provides low-threshold testing procedures and implementation of an exercise program without the need for expert knowledge. This fact is also relevant in times after the pandemic constraints, because supplementation of the traditional treatments with the best possible physical exercise program is always challenging for nursing assistants who do not have the special training to deliver an individualized exercise program. The InCoPE-App will allow nursing assistants to regularly test the physical and cognitive performance of each individual and to subsequently train people with dementia on that basis. To the best of our knowledge, there is currently no app available that applies a similar approach in a nursing home setting. The InCoPE app is expected to provide nursing assistants a means to provide their residents with dementia a usable and feasible individualized exercise program that may increase physical and cognitive performance as well as quality of life. This may in turn have a positive impact on disease progression and nursing care.

## Abbreviations

6MWT: 6-meter walk test
CG: control group
CST: chair stand test
FES-I: Falls Efficacy Scale International Version
FICSIT-4: Frailty and Injuries: Cooperative Studies of Intervention Techniques-subtest 4
IG: intervention group
InCoPE: Individualized Cognitive and Physical Exercise
MMSE: Mini-Mental State Examination
PSSUQ: Post-Study System Usability Questionnaire
QOL-AD: Quality of Life–Alzheimer’s Disease
TUG: Timed Up & Go
